# Clinical significance of circulating tumour cells and Ki-67 in renal cell carcinoma

**DOI:** 10.1186/s12957-021-02268-5

**Published:** 2021-05-25

**Authors:** Jinbo Song, Zhe Yu, Bingqi Dong, Mingkai Zhu, Xiaofeng Guo, Yongkang Ma, Shiming Zhao, Tiejun Yang

**Affiliations:** Department of Urology, The Affiliated Cancer Hospital of Zhengzhou University, Henan Cancer Hospital, Zhengzhou, China

**Keywords:** Circulating tumour cells (CTCs), Circulating tumour microemboli (CTM), Perioperative period, Renal cell carcinoma, Ki-67

## Abstract

**Background:**

Renal cell carcinoma (RCC) is a common malignant tumour of the genitourinary system. We aimed to analyse the potential value of metastasis-related biomarkers, circulating tumour cells (CTCs) and the proliferative marker Ki-67 in the diagnosis of RCC.

**Methods:**

Data from 24 laparoscopic radical nephrectomies (RNs) and 17 laparoscopic partial nephrectomies (PNs) were collected in 2018. The numbers and positive rates of CTCs and circulating tumour microemboli (CTM) in the peripheral blood were obtained at three different time points: just before surgery, immediately after surgery and 1 week after surgery. Ki-67 protein expression was evaluated in the RCC tissue by immunohistochemistry.

**Results:**

Except for the statistically significant association between the preoperative CTC counts and tumour size, no association between the number and positive rate of perioperative CTCs and clinicopathological features was found. The CTC counts gradually decreased during the perioperative period, and at 1 week after surgery, they were significantly lower than those before surgery. High Ki-67 expression was significantly positively correlated with preoperative CTC counts. In addition, Ki-67 expression was higher in the high CTC group (≥ 5 CTCs).

**Conclusion:**

Our results suggest that surgical nephrectomy is associated with a decrease in CTC counts in RCC patients. CTCs can act as a potential biomarker for the diagnosis and prognosis of RCC. A careful and sufficient long-term follow-up is needed for patients with high preoperative CTC counts.

**Supplementary Information:**

The online version contains supplementary material available at 10.1186/s12957-021-02268-5.

## Introduction

Renal cell carcinoma (RCC) is one of the most prevalent urological tumours. Its incidence rate is approximately 2% to 3% and continues to increase [1]. It is a serious threat to the health and life of patients. Because of the poor response to radiotherapy and chemotherapy, surgery (radical nephrectomy, RN; and partial nephrectomy, PN) is the first treatment option for RCC [2]. However, postoperative recurrence and metastasis still occur in up to 20% to 40% of RCC patients [2, 3]. Transcirculatory metastasis is the most important pathway for the formation of RCC metastasis foci. Circulating tumour cells (CTCs) are defined as tumour cells shedding from the primary tumour site or metastases into the peripheral blood and are also called circulating tumour microemboli (CTM) when they present as a cluster of cells. They may attach to and grow in distant organs and have long been considered a marker of tumour invasiveness [4, 5].

There are several methods for the precise diagnosis of RCC. In addition to pathological diagnosis, various molecular biomarkers enable the early detection of RCC or evaluation of its progression and prognosis [6–8]. Among these molecules, Ki-67 has been considered an effective diagnostic marker for a variety of cancers, including RCC [9–12]. Previous studies have indicated that the tumour proliferation rate is an important prognostic indicator with higher tumour spread rates leading to worse patient outcomes [13]. Ki-67, a nuclear protein that is associated with ribosomal RNA synthesis and may be necessary for proliferating cells, contributes to the enhanced proliferative activity of intrinsic cell populations in malignant tumours [14]. Therefore, Ki-67 has been considered a biomarker of RCC that could be used in routine clinical practice [11, 12, 15, 16]. In view of its clinical significance, Ki-67 has been widely used as a diagnostic approach for assessing tumour malignancies [17, 18].

The aim of the present study was to detect changes in the CTC and CTM counts and positive rates in the peripheral blood of patients with RCC during the perioperative period and to explore their correlation with clinical and pathological features (age, sex, tumour location, type, tumour staging and so on). In particular, we analysed the correlation between CTCs and the Ki-67 index and the prognostic value of CTCs in RCC.

## Materials and methods

### Clinical specimens

A total of 50 patients with kidney tumours who had received resection at The Affiliated Cancer Hospital of Zhengzhou University between January 2018 and December 2018 were enrolled in this study. The inclusion criteria were as follows: (1) a definitive pathological diagnosis of primary RCC, (2) received surgical resection, defined as complete removal of the macroscopic tumour, (3) margin-negative R0 resection, (4) no prior anticancer treatment, and (5) aged between 18 and 80 years. The exclusion criteria were as follows: (1) incomplete clinical evaluations; (2) a history of any urological surgery; (3) receiving any preoperative treatment, benign final pathology or upper tract urothelial cell carcinoma; and (4) having other active or preexisting malignancies. Finally, 41 patients were included. All surgical procedures were performed by the same surgeon in this department. This study was approved by the Ethical Committee of The Affiliated Cancer Hospital of Zhengzhou University, and all patients provided written informed consent.

### Collection and detection of CTCs

**Fig. 1:**
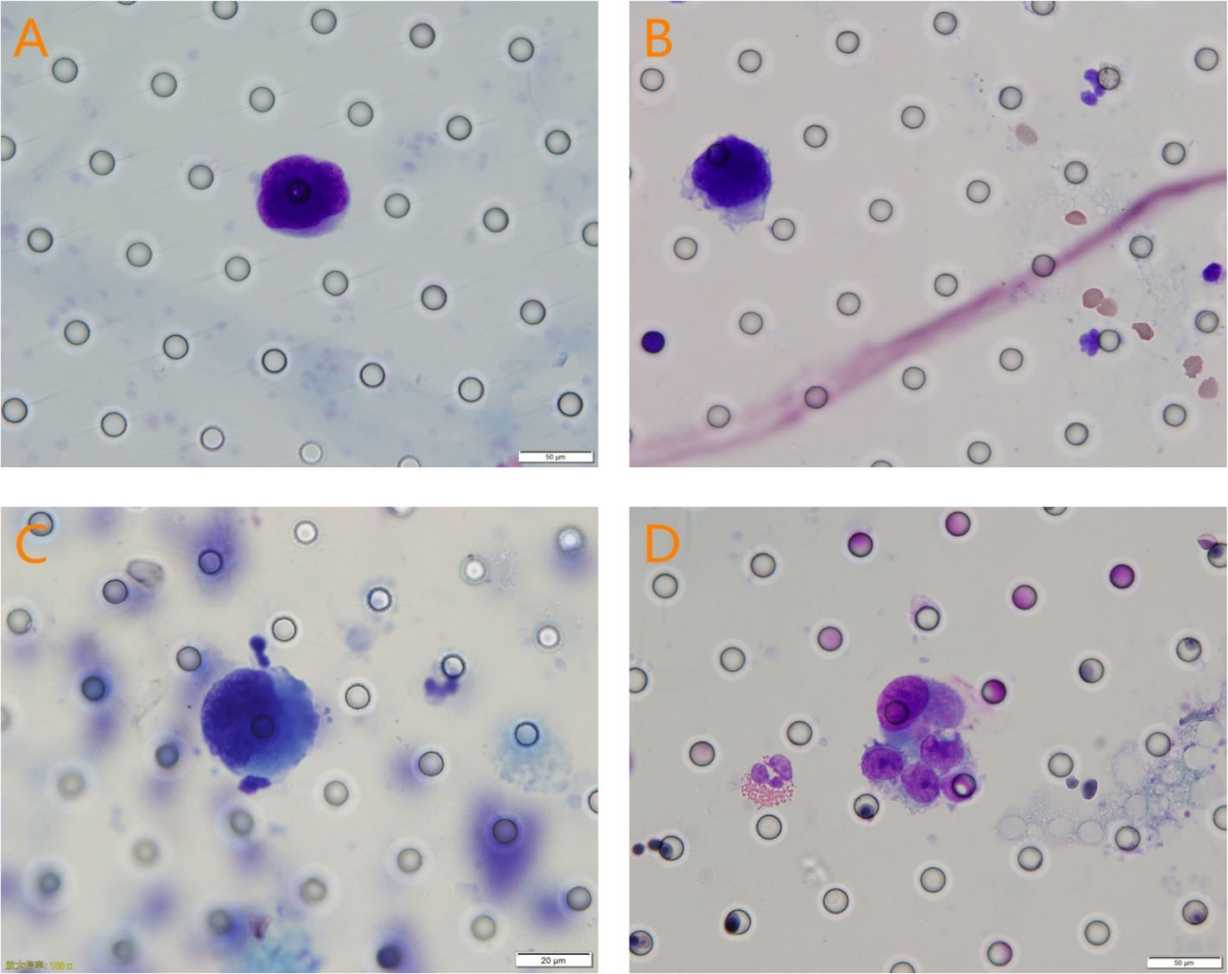
Cell morphology analysis of CTCs/CTM detected in the peripheral blood of RCC patients: **A**–**D** Cells were separated by the ISET method, and the cells of RCC patients were stained with Diff-Quik, showing malignant cell characteristics. CTCs (**A**–**C**): (1) nucleocytoplasmic ratio > 0.8, abnormally large nucleoli; (2) cell nucleus diameter > 18 mm, irregular nucleus shape; and (3) thickened jagged and wrinkled nuclear membrane, uneven staining, and lateral shift of chromatin. CTM (**D**): presence of the accumulation of tumour cells (≥ 3)

Peripheral blood was collected from RCC patients before, immediately after and 1 week after surgery. The first 1 mL of peripheral blood was discarded to avoid sample contamination. A 5-mL sample of total peripheral blood from each patient was stored in anticoagulant vessels with ethylenediaminetetraacetic acid (EDTA) and then mixed up and down slowly 8 times. After collection, the specimens were stored at room temperature and processed within 2 h. Otherwise, they were stored in a refrigerator at 4 °C for no more than 24 h. Samples were reheated at room temperature for at least 30 min before processing. As previously described, CTCs/CTM were analysed using the CTC-BIOPSY system (Youzhiyou, Wuhan, China), a semi-automatic CTC detection system based on membrane filtration separation technology (isolation by size of epithelial tumour cells, ISET) [19, 20]. Briefly, 5-mL specimens were diluted to 8 mL using buffer containing 0.2% formaldehyde and then transferred to a filtration membrane with a pore diameter of 8 μm. The sample was separated semi-automatically by the instrument, and then the cells remaining on the filter membrane were distinguished by Diff-Quik staining. CTCs/CTM were defined based on the following morphological standards [21]: (1) nucleocytoplasmic ratio > 0.8, nucleoli abnormally large; (2) cell nucleus diameter > 18 mm, nucleus irregularly shaped; (3) nuclear membrane thickened, wrinkled, non-uniform stained, and chromatin shifted laterally; and (4) CTM: 3 or more CTC aggregates. All candidate CTCs/CTM were blindly reviewed and identified independently by three senior cytopathologists. The staining results are shown in Fig 1.

### Immunohistochemical (IHC) staining of Ki-67

**Fig. 2:**
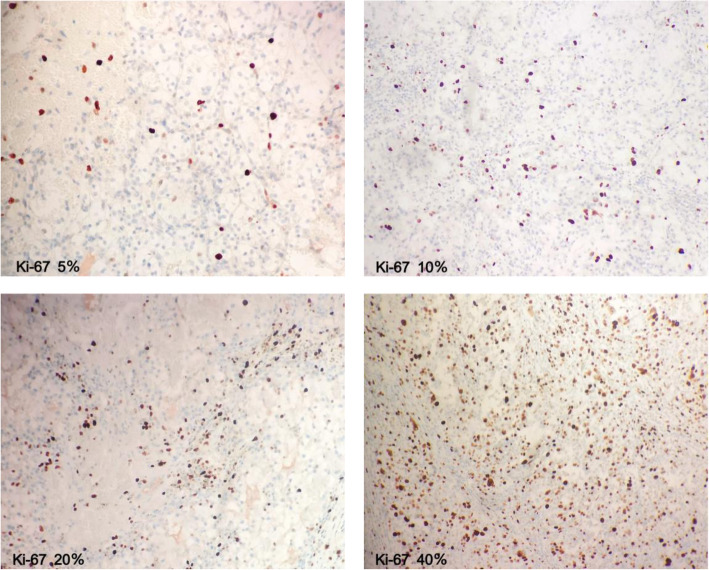
Ki-67 expression in RCC patients by immunohistochemistry. Brown nuclear staining highlights Ki-67-positive tumour cells

Tissue samples from representative areas were selected from RCC specimens obtained by surgery, fixed with formalin tissue fluid and embedded in paraffin. Samples were deparaffinized with xylene, repaired in the repair solution under high pressure and high temperature for 2 min, cooled to room temperature and washed with phosphate-buffered saline (PBS) 3 times for 15 min. The samples were then treated with 3% H_2_O_2_ for 10 min and washed with PBS as before. A primary antibody raised against Ki-67 (1:100, Gene Tech; Shanghai, China) was then added for immunohistochemical detection, and the sample was washed with PBS 3 times. The samples were incubated for 30 min at 37 °C by using a horseradish peroxidase*-*labelled secondary antibody. Diaminobenzidine (DAB) was used as the chromogen. Samples were washed with water for 10 min, stained with haematoxylin again and washed until colourless. The stained slides were examined by two independent pathologists who were completely blinded to the clinical data. Cells labelled with the Ki-67 antibody displayed a nuclear staining pattern. The score was assigned according to the average extent of immunoexpression (0–100% of cells stained). The staining results are shown in Fig 2.

### Statistical analysis

All statistical analyses were performed using GraphPad Prism 7.0 (GraphPad Software, CA, USA) and SPSS 22.0 (SPSS, Chicago, USA) statistical software. The results are presented as the mean ± standard deviation (M ± SD). Student’s t test and one-way analysis of variance (ANOVA) with Tukey’s test were used to compare continuous variables, and the chi-square test or Fisher’s exact test was used to compare categorical variables. The Wilcoxon two-sample test was used to compare non-normally distributed variables. Differences were considered statistically significant when the *P* value was < 0.05.

## Results

### Patient characteristics

Among the 41 patients included, there were 22 males and 19 females. The age ranged from 20 to 76 years (53.0 ± 12.1), and 34% of patients were older than 60 years. The pathological type has 37 cases of clear cell carcinoma, 4 cases of non-clear cell carcinoma (3 cases of papillary cell carcinoma and 1 case of chromophobe cell carcinoma); in the surgical method, 17 patients received PN, and 24 patients received RN. The baseline characteristics of the patients are shown in Table 1.

**Table 1:** The baseline characteristics of 41 RCC patients

Parameter	No. of patients	Percentage
Sex		
Male	22	53.7
Female	19	42.3
Age		
≤ 60 years	27	65.9
> 60 years	14	34.1
Tumour location		
Upper	8	19.5
Middle	16	39.0
Lower	17	41.5
Surgical methods		
Partial nephrectomy	17	41.5
Radical nephrectomy	24	58.5
Pathological type		
Clear cell carcinoma	37	90.2
Non-clear cell carcinoma	4	9.8
Fuhrman grade		
G1	14	34.1
G2	18	43.9
G3	6	14.6
G4	3	7.3

### Preoperative intraoperative and postoperative positive rates of CTC/CTM and CTC/CTM counts

**Fig. 3:**
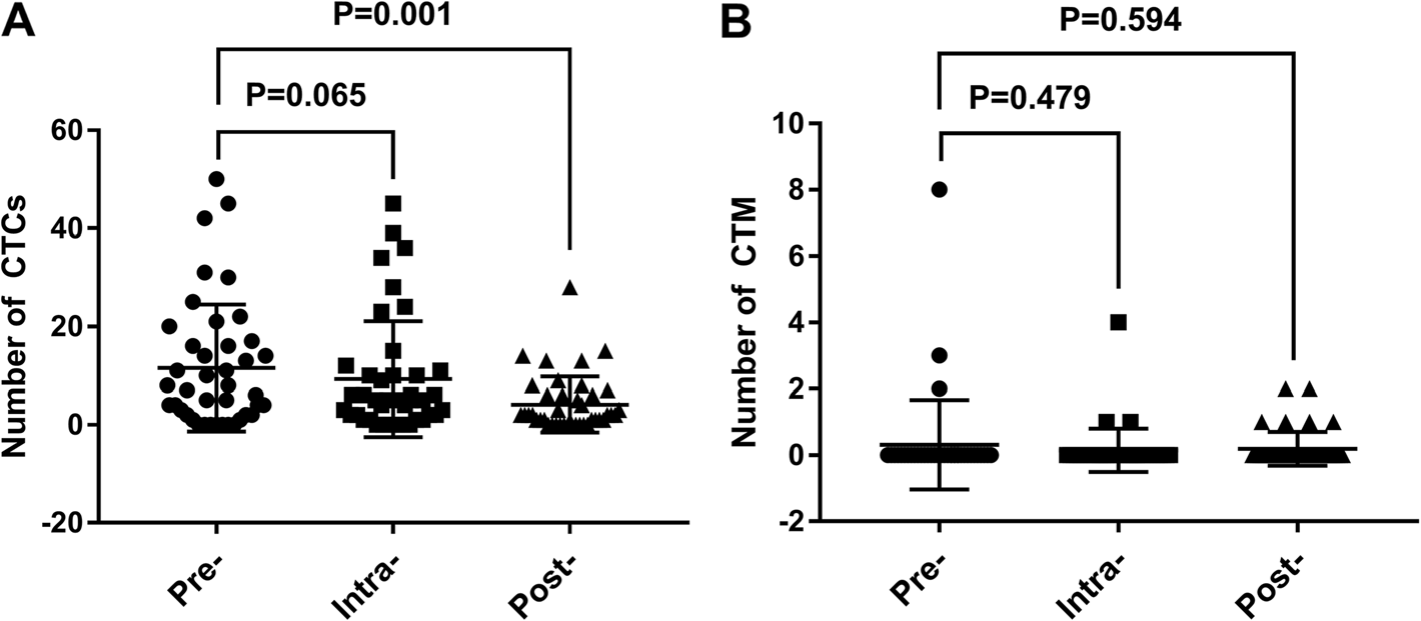
**A** Peripheral blood CTC counts in RCC patients before surgery, immediately after surgery and 1 week after surgery. **B** Peripheral blood CTM counts in RCC patients before surgery, immediately after surgery and 1 week after surgery. *Pre*, preoperation (before surgery); *Intra*, intraoperation (immediately after surgery); *Post*, postoperation (1 week after surgery)

The positive rates of CTCs in the peripheral blood of RCC patients before, immediately after and 1 week after surgery were 82.9%, 85.4% and 73.2%, respectively. The positive rate of CTCs 1 week after surgery was lower than that before surgery, but the difference was not statistically significant (*P* > 0.05); the CTC counts were 11.56 ± 12.92/5 mL, 9.29 ± 11.79/5 mL and 4.12 ± 5.71/5 mL, respectively. The CTC counts 1 week after surgery were significantly lower than those before surgery (*P* = 0.001) (Fig 3A). The detection rate of perioperative CTM in RCC patients was low. There was no statistically significant difference between the perioperative CTM counts or positive rates (Fig 3B). Thus, we did not perform subsequent analyses to determine whether the change in perioperative CTM affects the diagnosis and prognosis of RCC. Compared with CTC counts before surgery, CTC counts decreased in 26 (63.4%) patients, increased in 11 (26.8%) patients, and were unchanged in 4 (9.8%) patients immediately after surgery and decreased in 30 (73.2%) patients, increased in 9 (21.9%) patients, and were unchanged in 2 (4.9%) patients 1 week after surgery.

### CTC counts and their association with the pathological features of RCC patients

**Fig. 4:**
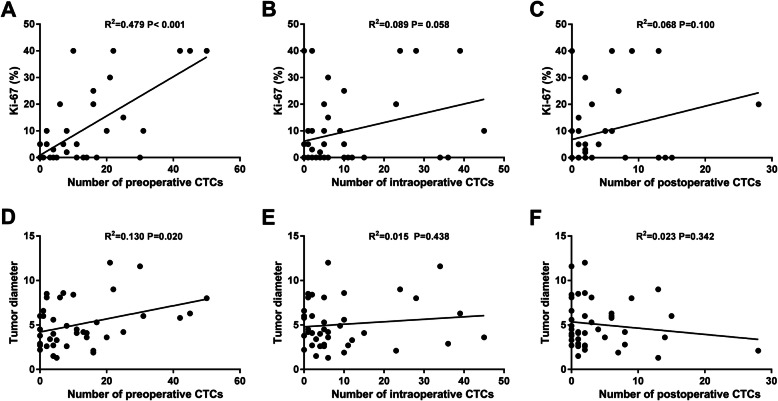
Correlation between the number of CTCs and Ki-67 and between the number of CTCs and tumour diameter. **A** Correlation between Ki-67 and the number of CTCs before surgery. **B** Correlation between Ki-67 and the number of CTCs immediately after surgery (number of intraoperative CTCs). **C** Correlation between Ki-67 and the number of CTCs 1 week after surgery. **D** Correlation between tumour diameter and the number of CTCs before surgery. **E** Correlation between tumour diameter and the number of CTCs immediately after surgery (number of intraoperative CTCs). **F** Correlation between tumour diameter and the number of CTCs 1 week after surgery

The correlations of CTC counts with the pathological features of patients before, immediately after and 1 week after surgery were analysed. As shown in Table 2, 17 patients had a tumour diameter 5 cm or more, 24 patients had a tumour diameter of less than 5 cm, and a significant correlation was observed between the tumour diameter and preoperative CTC counts (17.12 ± 16.94 vs. 7.13 ± 7.67; P = 0.018). Accordingly, linear regression analysis demonstrated significant positive correlations between preoperative CTC counts and tumour diameter (*P* = 0.020; Fig 4D). However, there was no significant difference immediately after surgery (*P* = 0.438; Fig 4E) or 1 week after surgery (*P* = 0.342; Fig 4F). Furthermore, the correlation analysis showed no correlation between the distribution of CTC-positive patients or CTC counts and clinical TNM stage (2017 AJCC renal cell carcinoma staging standard) before, immediately after and 1 week after surgery (Table 2, Supplementary table 1 and Supplementary table 2).

**Table 2:** The correlation of CTC counts with pathological features of patients before surgery

Pathological features	Cases (*n* = 41)	CTC counts (M ± SD)	*P* value	CTCs, n (%)		*P* value
				Negative (*n* = 7)	Positive (*n* = 34)	
Tumour size			0.018*			0.512
< 5 cm	24	7.13 ± 7.67		4 (16.7)	20 (83.3)	
≥ 5 cm	17	17.12 ± 16.94		3 (17.6)	14 (82.4)	
T-staging			0.621			0.433
T1-2	36	12.06 ± 14.08		7 (19.4)	29 (80.6)	
T3-4	5	9.50 ± 6.37		0 (0.0)	5 (100.0)	
N-staging			0.089			0.614
N0	38	11.92 ± 13.34		7 (18.4)	31 (71.6)	
N1	3	7.00 ± 3.00		0 (0.0)	3 (100.0)	
M-staging			0.354			0.517
M0	37	11.70 ± 13.38		7(18.9)	30(71.1)	
M1	4	10.25 ± 8.66		0 (0.0)	4 (100.0)	
AJCC staging			0.943			0.202
I	29	11.75 ± 14.36		7 (24.1)	22 (75.9)	
II	4	14.25 ± 13.52		0 (0.0)	4 (100.0)	
III	4	8.75 ± 4.27		0 (0.0)	4 (100.0)	
IV	4	10.25 ± 8.66		0 (0.0)	4 (100.0)	

^*^P < 0.05, the difference was statistically significant

*AJCC*, American Joint Committee on Cancer

TNM stage: 2017 AJCC renal cell carcinoma staging standard. *T*, tumor; *N*, lymph node; *M*, metastasis

As shown in Table 3, the patients were divided according to the surgical approach: 24 received laparoscopic RN, and 17 received laparoscopic PN. The changes in perioperative CTC counts were compared between the two groups, and the difference was not statistically significant (*P* > 0.05). However, the positive rate of peripheral blood CTCs in the RN group before surgery was significantly higher than that in the PN group (*P* = 0.014).

**Table 3:** The counts and positive rates of CTCs with different surgical approaches

Groups	Before surgery		Immediately after surgery		One week after surgery	
	Positive (%)	CTC counts	Positive (%)	CTC counts	Positive (%)	CTC counts
PN(*n* = 17)	11 (64.71)	10.29 ± 9.67	12 (70.59)	10.18 ± 11.32	13 (76.47)	5.00 ± 5.18
RN(*n* = 24)	23 (95.83)	12.46 ± 14.34	23 (95.83)	8.79 ± 7.76	17 (70.83)	3.50 ± 4.22
*P* value	0.014^*^	0.603	0.066	0.728	0.965	0.415

^*^P < 0.05, the difference was statistically significant

Then, using a preoperative CTC count of 5 as the cut-off value, 41 RCC patients were divided into a high CTC group (24 patients with ≥ 5 CTCs) and a low CTC group (17 patients with < 5 CTCs). There was no statistically significant difference in the change in CTCs between the two groups immediately after surgery (*P* = 0.885; Table 4). However, in the high CTC group, CTC counts decreased in 21 (87.5%) patients 1 week after surgery. Compared with the low CTC group (52.9%), the difference was statistically significant (*P* = 0.014; Table 4).

**Table 4:** The numbers of patients in different CTC groups who experienced changes in CTC counts during the perioperative period

	CTCs < 5, n (%) (*n* = 17 before surgery)		CTCs ≥ 5, n (%) (*n* = 24 before surgery)		*P* value
	Decreased	No alteration	Decreased	No alteration	
Immediately after surgery	11 (64.7)	6 (35.3)	15 (62.5)	9 (37.5)	0.885
One week after surgery	9 (52.9)	8 (47.1)	21 (87.5)	3 (12.5)	0.014*

^*^P < 0.05, the difference was statistically significant

### Correlation between CTC counts and Ki-67 expression in patients with RCC

According to the results of IHC staining, the patients were divided into three groups based on the Ki-67 index: those with an index less than 10% (26 patients), those with an index between 10 and 20% (8 patients), and those with an index of 21% or more (7 patients). Tumours with a Ki-67 index of 10% or more were considered to be highly proliferative. Based on this criterion, 15 of the 41 patients showed high proliferative potential. As shown in Table 5, the results suggested that the proportion of RCC patients with high CTC levels (≥ 5 CTCs) was higher in the high proliferation group than in the low proliferation group before surgery and 1 week after surgery. Moreover, the positive rate of preoperative CTCs in the high proliferation group was significantly higher than that in the low proliferation group (*P* = 0.035). However, the difference between them was not statistically significant immediately after surgery. Linear regression analysis demonstrated significant positive correlations between preoperative CTC counts and the Ki-67 index (*P* < 0.001; Fig 4A). However, no significant correlations were observed between the intra- and postoperative CTC counts and Ki-67 index (Fig 4B and C).

**Table 5:** The correlation between CTC counts and the Ki-67 index during the perioperative period

Before surgery		CTC counts, n		*P* value	CTCs, n (%)		*P* value
		< 5 (*n* = 17)	≥5 (*n* = 24)		Negative (*n* = 7)	Positive (*n* = 34)	
Ki-67 index	< 10 (*n* = 26)	15	11	0.012*	7(26.9)	19(73.1)	0.114
	10–20 (*n* = 8)	2	6		0(0.0)	8(100.0)	
	21–40 (*n* = 7)	0	7		0(0.0)	7(100.0)	
	Low proliferation (*n* = 26)	15	11	0.005*	7(26.9)	19(73.1)	0.035*
	High proliferation (*n* = 15)	2	13		0(0.0)	15(100.0)	
Immediately after surgery		CTC counts, n		*P* value	CTCs, n (%)		*P* value
		< 5 (*n* = 18)	≥ 5 (*n* = 23)		Negative (*n* = 6)	Positive (*n* = 35)	
Ki-67 index	< 10 (n = 26)	14	12	0.288	5 (19.2)	21 (80.8)	0.583
	10–20 (*n* = 8)	2	6		0 (0.0)	8 (100.0)	
	21–40 (*n* = 7)	2	5		1 (14.3)	6 (85.7)	
	Low proliferation (*n* = 26)	14	12	0.091	5 (19.2)	21 (80.8)	0.388
	High proliferation (*n* = 15)	4	11		1 (6.67)	14 (93.3)	
One week after surgery		CTC counts, n		*P* value	CTCs, n (%)		*P* value
		< 5 (*n* = 28)	≥ 5 (*n* = 13)		Negative (*n* = 11)	Positive (*n* = 30)	
Ki-67 index	< 10 (*n* = 26)	21	5	0.028*	7 (26.9)	19 (73.1)	0.612
	10–20 (*n* = 8)	5	3		3 (37.5)	5 (62.5)	
	21–40 (*n* = 7)	2	5		1 (14.3)	6 (85.7)	
	Low proliferation (*n* = 26)	21	5	0.038*	7 (26.9)	19 (73.1)	0.719
	High proliferation (*n* = 15)	7	8		4 (26.7)	11 (73.3)	

^*^P < 0.05, the difference was statistically significant

## Discussion

The detection of CTCs provides a new powerful tool to evaluate tumour load and invasiveness. In recent years, an increasing number of studies have been conducted on CTCs in RCC, which play an important role in the detection of early recurrence and metastasis [22]. Our results showed that preoperative CTC counts were higher in the tumour size ≥ 5 cm group and decreased after surgery. In addition, preoperative CTC counts were correlated with the proliferation marker Ki-67.

At present, there are few studies on CTCs in renal cell carcinoma. Here, we determined changes in perioperative CTC counts and positive rates and evaluated whether they were correlated with clinicopathological features. The statistical analysis demonstrated that only tumour diameter affected preoperative CTC counts. Our findings are not identical to those of previous studies that found that the TNM stage is related to the positive rate and level of CTCs [23, 24]. One reason could be that most patients were diagnosed with early clinical TNM stage disease.

Surgical nephrectomy is associated with a decrease in CTC counts. Our results showed that the mean level of CTCs gradually decreased during the perioperative period. Some studies have shown that in the surgical treatment of non-small-cell lung cancer and prostate cancer, the number of CTCs may decrease due to an invasive operation, leading to the blood-derived spillover of cancer cells [25, 26]. Zhang et al. showed that the levels of CTCs in breast cancer patients were higher on the 3rd day after surgery than before surgery, but decreased significantly on the 7th day after surgery [27]. An invasive operation might result in a transient increase in CTCs due to compressing the tumour. Overall, our findings are consistent with those of previous studies, and complete removal of the tumour will reduce CTC counts after surgery.

RN and PN are the main surgical approaches for renal cell carcinoma. According to the literature, different surgical methods may affect perioperative CTC counts. Haga et al. compared four surgical approaches: laparoscopic RN, laparoscopic PN, open RN and open PN. They found that open RN resulted in significantly higher postoperative CTC counts than laparoscopic RN or open or laparoscopic PN [28]. Our data showed a tendency for the RN group to have a higher positive rate of CTCs and more preoperative CTC counts than the PN group. The reason may be that patients had larger tumour diameters and more advanced TNM stages, which resulted in higher preoperative CTC counts and positive rates in the RN group. However, the perioperative change in the positive rate of CTCs or CTC counts did not differ significantly among the surgical methods. Laparoscopic surgery was performed on all patients in this study. Fine manipulation could be more possible in laparoscopic kidney surgery than in open surgery. Thus, laparoscopic kidney surgery might be preferable for preventing the blood-derived spillover of cancer cells.

Do preoperative CTC counts affect the change in CTCs after surgery? At present, there is no uniform definition of CTC positivity [29]. Some studies have reported that ≥ 5 CTCs is an independent risk factor for the recurrence and metastasis of breast cancer and non-small-cell lung cancer [27, 30]. Using a CTC count of 5 as the cut-off value, we demonstrated that more patients in the high CTC group showed decreased CTC counts 1 week after surgery than the low CTC group. To some extent, the high CTC group may receive extra benefit from surgical treatment.

Ki-67 is a nuclear antigen that is present in almost all human malignancies. A growing body of research on lymphomas, bladder cancer, colorectal cancer and gastric cancer has shown that the overexpression of Ki-67 is associated with tumour cell growth, biological aggressiveness and the prognosis of these malignancies [31–34]. Moreover, in RCC, the Ki-67 index was found to be positively associated with an advanced tumour stage and grade and provide an additional prognostic indication of biological aggressiveness [35, 36]. Tollefson et al. reported that patients with high Ki-67 expression were 68% more likely to die from RCC [37]. Then, we analysed the correlation between CTC counts and the Ki-67 index and evaluated the prognostic value of CTCs in RCC. Our results indicated that high Ki-67 expression was significantly positively correlated with the absolute number of preoperative CTCs using linear regression analyses. In addition, Ki-67 expression was higher in the high CTC group (≥ 5 CTCs). Therefore, ≥ 5 CTCs may be a prognostic indicator of renal cell carcinoma.

Compared with immunohistochemical staining, CTC detection is much more expensive. However, immunohistochemical staining can determine only the degree of malignancy of RCC patients after surgery. As a non-invasive detection method, we can observe dynamic changes in a patient’s CTCs during the perioperative period. Wang et al. dynamically observed the CTCs of 69 RCC patients before and after surgery. They found that the number of CTCs at 12 months after surgery was significantly higher than that before surgery and 6 months after surgery in metastatic patients [38]. They concluded that the recurrence or metastasis of RCC was probably related to the variation trend of CTCs. Recently, Nagaya et al. described a metastatic RCC patient, whose CTC counts rapidly increased after sunitinib treatment and then gradually decreased [39]. This result suggested that CTCs, as a promising biomarker, may be helpful for promptly monitoring the treatment response.

The methods used to detect CTCs in this study should be noted. The CellSearch system, which analyses CTCs by detecting epithelial cellular adhesion molecule (EpCAM) expression in individual tumours, was approved by the FDA as a CTC detection platform. However, the expression of EpCAM in RCC is not high [40], so this method has a low detection rate of CTCs in RCC [41]. In this study, a semi-automatic CTC detection system based on ISET technology, namely the CTC-BIOPSY device, was used to analyse CTCs. Compared with the CellSearch system, ISET has a higher detection rate for CTCs in RCC and more advantages in detecting renal cancer CTCs with low EpCAM expression [23]. In recent studies, markers of the G250 antigen or CA9 combined with CD147 have shown good prospects in the detection of CTCs in RCC patients [42–44].

A few limitations of this study need to be considered. First, to execute the inclusion and exclusion criteria in strict rotation, the number of patients enrolled in this study was small. Second, although preoperative CTC counts were correlated with the proliferative marker Ki-67 in the current study, the prognostic value and predictive impact of CTCs in the perioperative period need more than several years of observation because the follow-up period was too short. Future studies with a longer postoperative follow-up are necessary to assess the clinical significance of perioperative CTC detection in the diagnosis and treatment of RCC.

## Conclusion

Our results showed the effect of surgical nephrectomy on CTCs in patients with RCC. Our findings supported that surgical treatment was the direct reason for the decrease in CTC counts in RCC patients. Our results also showed a high association between CTC counts and the proliferation marker Ki-67 further confirming the potential of the CTCs as a diagnostic and prognostic biomarker of RCC. It is necessary to detect CTCs in RCC patients during the perioperative period, especially for those with a tumour ≥ 5cm in size. A careful and sufficient long-term follow-up is needed for patients with high preoperative CTC counts (≥ 5 CTCs). In future studies, the combination of CTC counts and the Ki-67 index or other biomarkers might provide better diagnostic accuracy and precision for RCC.

## Supplementary Information

**Additional file 1: Supplementary table 1.** The correlation of CTC counts with the pathological features of patients immediately after surgery

**Additional file 2: Supplementary table 2.** The correlation of CTC counts with the pathological features of patients one week after surgery

## Data Availability

All data generated or analysed during this study are included in this published article (and its supplementary information files).
