# Supplementary material for: Clinical significance of circulating tumour cells and Ki-67 in renal cell carcinoma
Source: World J Surg Oncol. 2021 May 25;19:156. doi: 10.1186/s12957-021-02268-5 (PMC8152311; doi:10.1186/s12957-021-02268-5)
Supplement: Supplementary file 1 — Additional file 1: Supplementary table 1. The correlation of CTC counts with the pathological features of patients immediately after surgery [file 12957_2021_2268_MOESM1_ESM.doc]

**Supplementary table 1** The correlation of CTC counts with the pathological features of patients immediately after surgery

| Pathological features | Cases  (n=41) | CTC counts  (M±SD) | *P-*value | CTCs, n (%) | | *P-*value |
| --- | --- | --- | --- | --- | --- | --- |
|  |  |  |  | Negative(n=6) | positive(n=35) |  |
| Tumor size |  |  | 0.812 |  |  | 0.137 |
| <5 cm | 24 | 8.92±11.14 |  | 4(16.7) | 20(83.3) |  |
| ≥5 cm | 17 | 9.82±12.97 |  | 2(11.8) | 15(88.2) |  |
| T-Staging |  |  | 0.784 |  |  | 0.985 |
| T1-2 | 36 | 9.54±12.64 |  | 6(16.7) | 30(83.3) |  |
| T3-4 | 5 | 8.25±7.85 |  | 0(0.0) | 5(100.0) |  |
| N-Staging |  |  | 0.231 |  |  | 0.519 |
| N0 | 38 | 9.63±12.15 |  | 6(15.8) | 32(84.2) |  |
| N1 | 3 | 5.00±4.36 |  | 0(0.0) | 3(100.0) |  |
| M-Staging |  |  | 0.354 |  |  | 0.731 |
| M0 | 37 | 9.08±13.38 |  | 6(16.2) | 31(83.8) |  |
| M1 | 4 | 11.25±1.34 |  | 0(0.0) | 4(100.0) |  |
| AJCC Staging |  |  | 0.829 |  |  | 0.905 |
| I | 29 | 9.10±12.57 |  | 6(20.7) | 23(79.3) |  |
| II | 4 | 12.75±14.64 |  | 0(0.0) | 4(100.0) |  |
| III | 4 | 5.25±3.59 |  | 0(0.0) | 4(100.0) |  |
| IV | 4 | 11.25±10.34 |  | 0(0.0) | 4(100.0) |  |

AJCC: American Joint Committe on cancer
