# Supplementary material for: Clinical significance of circulating tumour cells and Ki-67 in renal cell carcinoma
Source: World J Surg Oncol. 2021 May 25;19:156. doi: 10.1186/s12957-021-02268-5 (PMC8152311; doi:10.1186/s12957-021-02268-5)
Supplement: Supplementary file 2 — Additional file 2: Supplementary table 2. The correlation of CTC counts with the pathological features of patients one week after surgery [file 12957_2021_2268_MOESM2_ESM.doc]

**Supplementary table 2** The correlation of CTC counts with the pathological features of patients one week after surgery

| Pathological features | Cases  (n=41) | CTC counts  (M±SD) | *P-*value | CTCs, n (%) | | *P-*value |
| --- | --- | --- | --- | --- | --- | --- |
|  |  |  |  | Negative(n=11) | positive(n=30) |  |
| Tumor size |  |  | 0.910 |  |  | 0.502 |
| <5 cm | 24 | 4.21±6.47 |  | 5(20.8) | 19(79.2) |  |
| ≥5 cm | 17 | 4.00±4.62 |  | 6(35.3) | 11(64.7) |  |
| T-Staging |  |  | 0.838 |  |  | 0.300 |
| T1-2 | 36 | 4.03±5.82 |  | 11(33.3) | 25(66.7) |  |
| T3-4 | 5 | 4.50±5.63 |  | 0(0.0) | 5(100.0) |  |
| N-Staging |  |  | 0.127 |  |  | 0.619 |
| N0 | 38 | 4.37±5.81 |  | 10(26.3) | 28(73.7) |  |
| N1 | 3 | 1.00±1.00 |  | 1(33.3) | 2(66.7) |  |
| M-Staging |  |  | 0.999 |  |  | 0.559 |
| M0 | 37 | 4.05±5.80 |  | 11(29.7) | 26(80.3) |  |
| M1 | 4 | 4.5±5.56 |  | 0(0.0) | 4(100.0) |  |
| AJCC Staging |  |  | 0.632 |  |  | 0.296 |
| I | 29 | 4.52±6.05 |  | 9(31.0) | 20(69.0) |  |
| II | 4 | 0.50±1.00 |  | 1(25.0) | 3(75.0) |  |
| III | 4 | 4.25±6.55 |  | 1(25.0) | 3(75.0) |  |
| IV | 4 | 4.75±5.56 |  | 0(0.0) | 4(100.0) |  |

AJCC: American Joint Committe on cancer
